# Fast and Parallel Detection of Four Ebola Virus Species on a Microfluidic-Chip-Based Portable Reverse Transcription Loop-Mediated Isothermal Amplification System

**DOI:** 10.3390/mi10110777

**Published:** 2019-11-14

**Authors:** Xue Lin, Xiangyu Jin, Bin Xu, Ruliang Wang, Rongxin Fu, Ya Su, Kai Jiang, Han Yang, Ying Lu, Yong Guo, Guoliang Huang

**Affiliations:** 1 Department of Biomedical Engineering, the School of Medicine, Tsinghua University, Beijing 100084, China; lin-x15@mails.tsinghua.edu.cn (X.L.); 18810913852@163.com (X.J.); jackwrl@163.com (R.W.); frx14@mails.tsinghua.edu.cn (R.F.); suya_1127@163.com (Y.S.); thujiangkai@foxmail.com (K.J.); yhanbupt@163.com (H.Y.); ying-lu@tsinghua.edu.cn (Y.L.); yongguo@tsinghua.edu.cn (Y.G.); 2 Department of Precision Instrument, Tsinghua University, Beijing 100084, China; dongjq@vip.sina.com; 3 National Engineering Research Center for Beijing Biochip Technology, Beijing 102206, China

**Keywords:** Ebola virus, detection, reverse transcription loop-mediated isothermal amplification, microfluidic chip

## Abstract

Considering the lack of official vaccines and medicines for Ebola virus infection, reliable diagnostic methods are necessary for the control of the outbreak and the spread of the disease. We developed a microfluidic-chip-based portable system for fast and parallel detection of four Ebola virus species. The system is based on reverse transcription loop-mediated isothermal amplification (RT-LAMP) and consists of four specific LAMP primers, a disc microfluidic chip, and a portable real-time fluorescence detector. It could specifically and parallelly distinguish four species of the Ebola virus after only one sampling, including the Zaire Ebola virus, the Sudan Ebola virus, the Bundibugyo Ebola virus, and the Tai Forest Ebola virus, without cross-contamination. The limit of detection was as small as 10 copies per reaction, while the total consumption of sample and reagent was 0.94 μL per reaction. The final results could be obtained in 50 min after one addition of sample and reagent mixture. This approach provides simplicity, high sensitivity, and multi-target parallel detection at a low cost, which could enable convenient and effective on-site detections of the Ebola virus in the outdoors, remote areas, and modern hospitals.

## 1. Introduction

Ebola virus is a filovirus containing a non-segmented, negative-sense, and single-stranded ribonucleic acid (RNA) genome and belongs to the family Filoviridae [1,2]. The genus Ebola virus includes four human pathogenic species, Zaire Ebola virus (EBOV), Sudan Ebola virus (SUDV), Bundibugyo Ebola virus (BDBV), and Tai Forest Ebola virus (TAFV) [3]. According to the World Health Organization (WHO), the average mortality rate of the EBOV case is approximately 50%. In the 2014–2016 outbreak in West Africa, 28,610 people were involved, and 39.5% of them lost their lives [4]. A new outbreak was declared in February 2019 in the towns of Butembo and Katwa in the Northeast area of the Democratic Republic of Congo. As of 15 July 2019, a total of 2512 cases were reported, including 2418 confirmed and 94 probable cases, of which 1676 died (overall fatality ratio: 67%). The WHO declared the Ebola virus disease in the Democratic Republic of Congo as a public health emergency of international concern on 17 July [5]. The prevention and the control of the Ebola virus is still a serious worldwide issue. The incubation period of the Ebola virus disease is 2 to 21 days, and the symptoms overlap with those of other more common endemic diseases, such as malaria, making it difficult to diagnose [6]. In addition, no proven vaccines or treatments for Ebola have been reported [4]. Hence, it is crucial to develop Ebola virus specific detection and fast diagnostic method to manage the spread of the disease on site.

Molecular detection methods mainly focus on antigens, antibodies, and nucleic acids related to Ebola virus. Enzyme-linked immunosorbent assay (ELISA) is extensively used to detect antigens and antibodies, but the relatively low dynamic range and sensitivity compared to nucleic acids detection restricts its practical use. Piraino et al. developed a microfluidic platform for digital immunoassay with high dynamic range [7]. The limit of detection (LOD) was improved three orders of magnitude but was still inappropriate for early detection of Ebola infection. Zang et al. used the nanoantenna-based biosensor and significantly improved the sensitivity [8]. The array was made of gold and was better suited to operate in a central lab. Amplification-free nucleic acid detection could provide a rapid and fast diagnosis test but needed complex structures or methods for a low LOD [9,10].

Six in-vitro diagnostic (IVD) assays for Ebola virus disease are accepted for procurement by the WHO; 50% of them are based on the reverse transcription polymerase chain reaction (RT-PCR) method [11]. Xpert Ebola Assay can be used to detect only the Zaire strain of the Ebola virus, though it has the advantages of automation and integration. Liferiver™-Ebola Virus (EBOV) Real Time RT-PCR Kit can differentiate four subtypes, but it needs a multi-step manual operation, which not only requires clean laboratory conditions but also can easily cause cross-contamination and infection. RealStar® Filovirus Screen RT-PCR Kit 1.0 can be used only for the detection and the differentiation of Ebola virus and Marburg virus [12]. The RT-PCR is a WHO-recommended IVD method owing to its high specificity. However, no automatic detection method for all Ebola virus species on site has been reported.

Loop-mediated isothermal amplification (LAMP) is a simple and sensitive nucleic acid amplification method under isothermal conditions. The absence of temperature cycle simplifies the supporting equipment [13]. Carter et al. developed a visually readable detection method based on RT-LAMP, which is convenient and simple, but the visual detection cannot be used to distinguish different concentrations [14]. Kurosaki et al. and Oloniniyi et al. used a real-time fluorescence detection platform (Genie III; OptiGene, West Sussex, UK) for monitoring and display [15,16]. The whole reaction volume is 25 μL, and thus it requires the same amount of reagents as in the laboratory. Moreover, all steps still need to be operated by experienced technicians, making them unsuitable for a field test in resource-limited areas such as Africa.

In this study, we propose an RT-LAMP assay for the detection of Ebola on site by using a microfluidic-chip-based portable system. The fluorescence signal was recorded and plotted in real time. After only one sampling, the reaction and the detection could be auto-completed in 50 min. Moreover, the chip-based portable system could distinguish four Ebola viruses in a small reaction volume of 0.94 μL compared to the traditional laboratory tube volume of 25 μL per reaction.

## 2. Materials and Methods 

### 2.1. Preparation of Ebola Virus RNAs

The template plasmid for EBOV was a clinical isolation and was kindly provided by Professor Shengqi Wang, Beijing Institute of Radiation Medicine, China. Artificial plasmids spanning the glycoprotein (GP) were synthesized for other species of the Ebola virus by Sangon Biotech (shanghai, China). The sequences were determined by evaluating the GP genes of SUDV (GenBank accession no. NC006432), BDBV (GenBank accession no. NC014373), and TAFV (GenBank accession no. NC014372). All related RNA sequences, available in the National Center for Biotechnology Information GenBank (Bethesda, MD, USA), were aligned by using DNAstar MegAlign (DNASTAR, Madison, USA) to identify conversed sequences.

### 2.2. LAMP Primer Design

LAMP assays require a set of six primers, two outer primers (F3 and B3), two inner primers [forward inner primer (FIP) and backward inner primer (BIP)], and two loop primers (forward loop primer (LF) and backward loop primer (LB)). The primers targeting the GP were designed with the LAMP primer design software Primer Explorer version 4 (https://primerexplorer.jp/elamp4.0.0/index.html). The specificities of these primers were verified by the Basic Local Alignment Search Tool (BLAST) analysis (Version 4, Bethesda, MD, USA). The sequences of the primers are listed in Table 1. All primers were synthesized by Sangon Biotech (Shanghai, China).

### 2.3. LAMP Reactions in an Eppendorf (EP) Tube and on a Microfluidic Chip

LAMP reactions were performed in both an eppendorf (EP) tube and on the microfluidic chip. In the EP tube, the LAMP amplification was performed with a WarmStart LAMP Kit (DNA&RNA) (New England BioLabs Inc., Beijing, China) according to the manufacturer’s instructions. The reaction mixture (volume/tube, 25 μL) contained 12.5 μL of WarmStart LAMP 2× Master Mix, 0.5 μL of a fluorescent dye (50×), 2.5 μL of LAMP primer mix (10×), 4.5 μL of dH_2_O, and 5 μL of synthesized RNA dilution. The LAMP and the fluorescence detection were performed by using a real-time thermo-cycler (CFX96, Bio-Rad, Hercules, CA, USA) at 65 °C for 60 min.

On the microfluidic chip, the reactions were performed with the same kit and proportions of the components, but the total volume per reaction was 0.94 μL. In addition, a portable real-time fluorescence detector was used, as described below.

### 2.4. Microfluidic Chip Design

**Figure 1:**
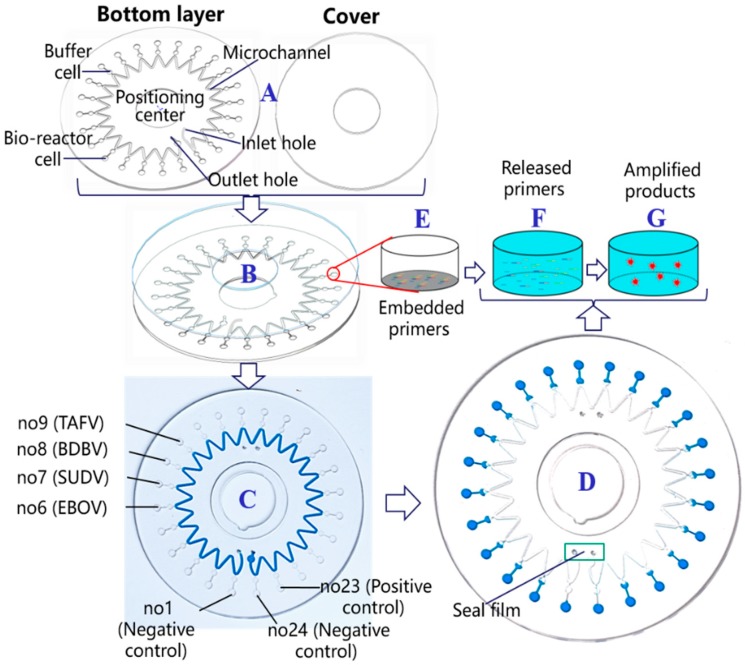
Schematic of the novel microfluidic chip for the parallel detection of four Ebola virus species. (**A**) Main composition of the microfluidic chip; (**B**) encapsulation of the chip; (**C**) sampling reaction mixture; (**D**) after the chip centrifugation; (**E**) embedded primers; (**F**) released primers at 50 °C; (**G**) amplified products binding with a fluorescent marker.

To reduce the consumptions of sample and reagent and simplify the operation, a novel microfluidic chip was designed, as shown in Figure 1. The microfluidic chip was composed of two layers, a bottom layer with a microstructure and a cover with double-sided adhesive tape. They were fabricated of polycarbonate (PC) materials by precision injection molding. After the ultrasonic cleaning and the centrifuge dripping of the bottom layer, the mixtures of primers and Sepharose CL-4B (Merck KGaA, Darmstadt, Germany) were spotted on the bio-reactor cells. The low-melting-point CL-4B did not participate in the amplification. The primers were released from the CL-4B when heated at 50 °C. After the alignment, the two layers were attached and sufficiently compressed by a press machine. Each chip was individually vacuum-packed and stored at −20 °C before use.

The structures of the bottom layer and the cover of the microfluidic chip are shown in Figure 1A, which consisted of a positioning center, inlet and outlet holes, a microchannel, 24 buffer cells, and 24 bio-reactor cells. Each of the six primers in Table 1 designed to identify one subtype of the Ebola virus were embedded together at the bottom of one bio-reactor cell by using the low-melting-point Sepharose CL-4B, as shown in Figure 1E. Primers corresponding to EBOV were embedded in the sixth, the tenth, and the fourteenth cells. Primers corresponding to SUDV were embedded in the seventh, the eleventh, and the fifteenth cells. Primers corresponding to BDBV were embedded in the eighth, the twelfth, and the sixteenth cells. Primers corresponding to TAFV were embedded in the ninth, the thirteenth, and the seventeenth cells. The positive quality control was embedded in the twenty-third cell; the others contained negative quality controls.

After the encapsulation of the chip by pressure bonding (Figure 1B), the reaction mixture including the sample was added to the chip by a micropipette through the inlet hole and filled the microchannel (Figure 1C). The inlet and the outlet holes were then sealed by a film sticker. The liquid was centrifugalized and evenly distributed to the 24 bio-reactor and the buffer cells. The reagents first filled the bio-reactor cells, and the buffer cells held extra reagents and bubbles, which ensured that all reaction cells had the same volume. With the help of the centrifugal force, adjacent buffer cells were not connected owing to the sine-shaped high-pressure air microchannel after the heating, which effectively avoided cross-contamination during the reactions. Each bio-reactor cell corresponded to a 0.94 μL tube. Twenty-four independent reactions could be simultaneously and parallelly performed after only one sampling (Figure 1D). Primers were preliminarily buried in bio-reactor cells with Sepharose CL-4B (Figure 1E) and released from agarose into the reaction mixture for amplification upon heating to 50 °C (Figure 1F). Subsequently, nucleic acid amplification occurred at 65 °C. The amplified products of the specific nucleic acid sequence were continuously generated. Simultaneously, the fluorescent marker EvaGreen automatically bound to the amplified products, which enabled real-time fluorescent signal detection by the portable system for the parallel identification of the four Ebola viruses, as shown in Figure 1G.

### 2.5. Microfluidic-Chip-Based Portable Detection System

**Figure 2:**
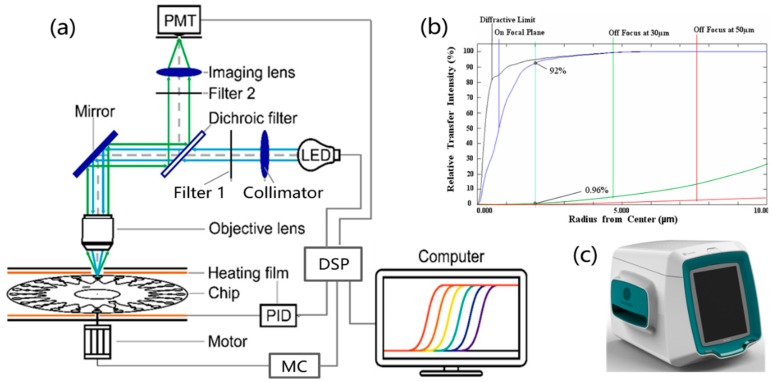
Portable real-time fluorescence detection system. (**a**) Schematic of the portable real-time fluorescence detection system; (**b**) fast Fourier transform (FFT) diffraction encircled energy diagram of its optical system; (**c**) photograph of the portable system.

To realize the parallel detection of multiple Ebola viruses on site, a microfluidic-chip-based portable real-time fluorescence detector was developed, as shown in Figure 2. The detector contained four parts, a heating module (including heating films and proportional integral derivative (PID) controller), a moving module (motor and multiple moving controller (MC)), a microprocessor (digital signal processor (DSP)), and an optics module. The heating films were maintained at distances of 0.25 mm from the microfluidic chip to enable fast and even two-sided heating. They were controlled by a PID controller connected to a computer; the temperature accuracy was 0.1 °C. The heating program could be set by the user, including the reaction temperature and the time. For the LAMP reaction, we incubated the chip at 65 °C for 60 min. The moving module could drive the chip for rotary scanning. The speed was also adjustable; we used a speed of 30 s per cycle. Thus, for each reaction cell, the florescent signal was monitored after every 30 s. In this manner, we could obtain real-time florescent signal graphs of the 24 reaction cells. Regarding the optics module, the excitation light source was a 1 W blue light-emitting diode (LED). The excitation light was collimated by a collimation lens, filtered by band-pass filter 1, reflected by a mirror, and focused to each bio-reactor cell by an objective lens. The florescent dye combined with double-stranded deoxyribonucleic acid in the reaction cells was excited and exhibited a green fluorescence. The fluorescence was reflected by the mirror and a dichroic filter, filtered by band-pass filter 2, focused by an imaging lens, and detected by a photo-multiplier (PMT) (Hamamatsu Photonics, Hamamatsu, Japan). In the optics module, novel confocal optical imaging was designed, where the fast Fourier transform (FFT) diffraction encircled energy indicated that the fluorescence collection to the bio-reactor cell on the chip was near the optical diffraction limit, as shown in Figure 2b. It could provide high sensitivity (>92%) to detect the amplified products of the trace nucleic acid in the bio-reactor cell of the microfluidic chip. A photograph of the portable system is shown in Figure 2c; the device is 32 cm × 30 cm × 26 cm in volume, and the weight is 8 kg. The device could run 8–10 h with a fully charged battery and could detect 8–10 samples per day.

The microfluidic-chip-based parallel detection of the four subtypes of the Ebola virus consisted mainly of the following steps: preparation of the LAMP reaction mixture, its addition to the microfluidic chip through the inlet hole by using a pipettor, sealing of the inlet and the outlet holes by a film sticker, and placement of the chip onto the pallet of the portable real-time fluorescence detector. The portable system then automatically carried out centrifugation, amplification, detection, and real-time display.

## 3. Results

### 3.1. Linearities of the Designed LAMP Primers

**Figure 3:**
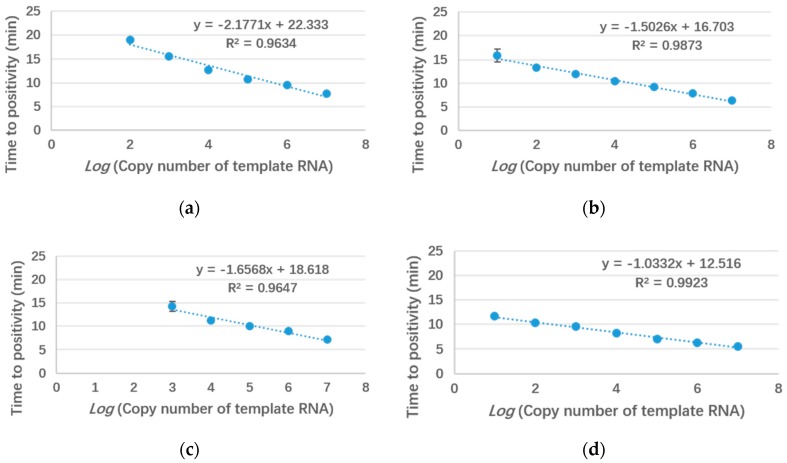
Linear fitting curves of the amplification times of the four subtypes of the Ebola virus to the template RNA concentration. (**a**) EBOV; (**b**) SUDV; (**c**) BDBV; (**d**) TAFV.

**Figure A1:**
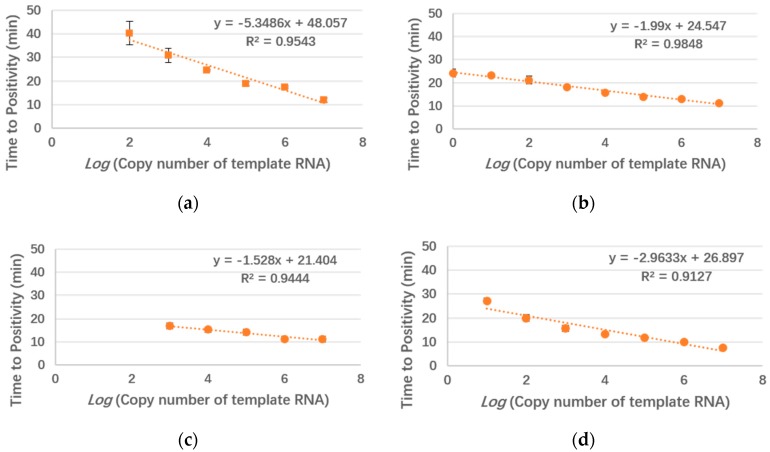
Linear fitting curves of the amplification times of the four subtypes of the Ebola virus to the template RNA concentration on the device. (**a**) EBOV; (**b**) SUDV; (**c**) BDBV; (**d**) TAFV.

To estimate the performances of the designed LAMP primers, serially diluted template plasmids were used in tube reactions (25 μL). Each reaction was repeated three times. The standard curves of the four subtypes are shown in Figure 3. Usually, time to positivity (Tp) is used as an index in LAMP reactions, defined as the time when the first derivative of the amplification curve exhibits an inflection point. Similar to the cycle threshold (Ct) in polymerase chain reaction (PCR), Tp indicates the initiation time of the exponential amplification in LAMP. Thus, smaller Tp values correspond to smaller reaction times. The tube assay could detect as few as 100 copies/μL of EBOV, 1000 copies/μL of BDBV, and 10 copies/μL of SUDV and TAFV. The coefficients of determination (R^2^) of all four subtypes were over 0.96, which indicated good linearities. For comparison, we also carried out a linearity test of the primers on our device and obtained a similar result (Figure A1).

### 3.2. Comparison of the Sensitivities in the Tube and on the Chip

**Figure 4:**
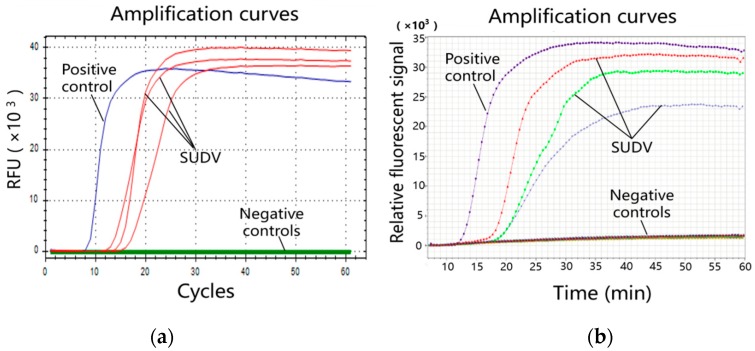
Comparison of the SUDV amplification curves in the tube and on the chip obtained by using templates of 10 copies per reaction. (**a**) LAMP amplification curves in the tubes—three repeated reactions (red lines) with a positive control (blue line) and negative controls (green lines); (**b**) LAMP amplification curves on the chip. The blue line corresponds to the twenty-third bio-reactor cell (positive control), while the red, the green, and the light blue lines correspond to the seventh, the eleventh, and the fifteenth cells (embedded with SUDV specific primers), respectively. The other cells contained negative controls.

Figure 4a shows LAMP amplification curves in EP tubes obtained by using SUDV templates of 10 copies per reaction. The Cts of the three repeated reactions (red lines) were 14.21, 15.62, and 17.65. The mean Ct was 15.83, while the standard deviation was 1.41. Figure 4b shows the amplification curves of all bio-reactor cells on the microfluidic chip obtained by using the same sample as in Figure 4a. The Tp values of the seventh, the eleventh, and the fifteenth cells (embedded with SUDV specific primers) were 15.20, 16.28, and 17.12, respectively. The mean TP was 16.20, while the standard deviation was 0.79. Compared to that for the reactions in the EP tubes, the on-chip amplification had a similar reaction time and small standard deviation.

Table 2 compares the sensitivities in the tube and on the chip. Serially diluted templates were used for each species. For EBOV, BDBV, and TAFV, the LODs of the chip method were equal to those of the tube method, while for SUDV, the chip method could be used to detect 1 copy/μL of template, which could not be achieved by using the tube method. This improvement may be attributed to the high sensitivity of the portable detection system.

### 3.3. Specificities of the Four Subtypes of the Ebola Virus on the Chip

As the chip had 24 bio-reactor cells, as many as 24 reactions could be simultaneously performed on the chip. For the Ebola virus detection, we needed to distinguish only the four species. For each species, we used three reaction cells to repeat the reactions to obtain more reliable detection results. The distribution of primers preloaded in the microfluidic chip was explained in the microfluidic chip design section. Templates (1000 copies/μL) of each species were tested to evaluate the specificities of the different species of the Ebola virus. The corresponding reaction cells and the positive control exhibited amplification signals, while the other negative controls did not exhibit such signals (Table 3). In addition, for a negative sample (water), only the positive control cell (no. 23) reacted. This indicated that the microfluidic chip designed for Ebola virus detection could differentiate the four species.

Table 3 shows the results of the specificity test for the four subtypes of the Ebola virus and a negative control on the chip, which indicated that our microfluidic-chip-based portable system could be reliably used for the parallel detection of the four subtypes of the Ebola virus without cross-contamination between different species.

## 4. Discussion

We developed a microfluidic-chip-based portable system for fast and parallel detection of the four Ebola virus species. By using the developed portable real-time fluorescence detector and a disc microfluidic chip, 24 independent nucleic acid amplifications could be simultaneously performed on a single chip after a single sample loading. The real-time fluorescent signals of the LAMP amplifications were efficiently collected near the optical diffraction limit by the portable detector, and amplification curves were dynamically displayed on the screen. The LODs of SUDV and TAFV were as low as 10 copies per reaction, and the total volume of sample and reagent was 0.94 μL per reaction. In this assay, the disc microfluidic chip was used to distinguish the four species of the Ebola virus. For each species, three parallel bio-reactor cells were used to improve the reliability of the results by excluding accidental errors. Studies on the use of LAMP to detect the Ebola virus [16,17] and microfluidic chips to diagnose Ebola [18,19] have been reported. However, to the best of our knowledge, this is the first report on the combination of LAMP and microfluidic techniques for the detection and the differentiation of the four Ebola virus subtypes.

The microfluidic-chip-based portable detection system has the following advantages.

(1) The specificities of the proposed assay are similar or even better than those of previously reported RT-PCR and RT-LAMP methods. The microfluidic chip could detect 100, 1, 1000, and 10 copies of EBOV, SUDV, BDBV, and TAFV per reaction, respectively. The RT-LAMP method has generally provided LODs of 10–100 copies/μL [15,17,20,21]. Considering the traditional reaction volume of RT-LAMP (25 μL), the total number of templates in the previous reports could be as low as 250–2500 copies of Ebola virus per reaction. Thus, our method is advantageous over the others in terms of specificity, and four species can be identified parallelly after only one sample addition. The comparison of the tube and the on-chip methods showed that the latter provided a higher performance for SUDV, likely owing to the high sensitivity of the portable detection system.
(2) The four species of the Ebola virus could be detected after only one sampling by a single chip. Notably, no extensive studies have been carried out on methods suitable to distinguish all four subtypes at a time. However, the correct classification is important for the diagnosis and the treatment of patients and is necessary for virus transmission and virology. In the proposed assay, we designed corresponding LAMP primers for each of the four species and repeated three reactions on one chip. According to the specificity test, the primers could be used to well differentiate the four subtypes without cross-contamination. As 24 bio-reactor cells were implemented on the microfluidic chip, primers for other endemic diseases could be added to develop a more complete chip for general virus detection.
(3) The chip method is better than the traditional tube method in terms of simplicity and convenience. First, the reaction volume on the chip (0.94 μL) is approximately 25 times smaller than that in the tube (recommended value for the LAMP kit: 25 μL), and thus the consumptions of reagents and samples can be largely decreased. Second, the assay requires only the portable real-time fluorescence detector and a microfluidic chip. Compared to the large-scale PCR amplification instruments, our system is smaller and portable and has similar or even better performances. In addition, the chip is more convenient for transportation and sample loading, as it does not require a specialized laboratory. The chemical reagents used in this article should be stored at −20 °C and transported in an ice box. The Ebola virus is relatively common in at-risk countries such as Guinea and Liberia. These locations have weak medical systems and inadequate equipment. Our assay can well satisfy the corresponding demands.

Current efforts are focused on the detection of four species of Ebola virus using RNA templates. RNAs extracted from commercial kits could be used in our system, and the following steps could be automatically completed. However, for an infectious virus such as Ebola, a fully integrated diagnosis is optimal. As a further development of this work, efforts are underway to combine sample preparation with our system for complete isolation from Ebola virus. 

## Figures and Tables

**Table 1:** Ebola virus species-specific loop-mediated isothermal amplification (LAMP) primer sets.

Species	Name	Sequences (5′-3′)
EBOV	Z-F3	ATCAATTGAGATCAGTTGGAC
	Z-B3	ACTTTGTGCACATACCGG
	Z-FIP	CCTGAAGCCCCATCTTTTAGTCACATGAATCTCGAGGGGAATGGA
	Z-BIP	TTATGAAGCTGGTGAATGGGCTGCTGGTAGACACTCACTC
	Z-LF	GCACGTCAGTTGCCAC
	Z-LB	GAAAACTGCTACAATCTTGAAATC
SUDV	S-F3	GCCTTAGTCTGTGGACTTAGG
	S-B3	GTGGCTCAATGCAACAATC
	S-FIP	GTCCGCAGCTCCGTTGTGCAACTTGCAAATGAAACAACTCA
	S-BIP	CCATACTCAATAGGAAGGCCATAGCCCAGGATCCTGCATGTC
	S-LF	CTTAAGAAAAGCTGCAGAGC
	S-LB	ATTTCCTTCTGCGACGAT
BDBV	B-F3	CCCAAAGTGGTGAACTACGA
	B-B3	AGCGCCTTCTTTGTGGAAAG
	B-FIP	CAGGGGCTTCAGGTAGGCATTCGGGAGTGGGCTGAAAACTG
	B-BIP	GGTGTAAGAGGCTTCCCTCGCAAACCTTCAGGACACGGC
	B-LF	GCTTTCTTGATGTCCAGGTTGTA
	B-LB	GTTATGTGCACAAGGTTTCTGGAAC
TAFV	T-F3	TAGAGGCACGGGACCATC
	T-B3	TTCTGGATCCTGTCAGGAGA
	T-FIP	GCAGTGTGCTGTTCTGGGAGTGAACACCACAGAAAGCCACG
	T-BIP	GCCAGTGCCATTCCAAGAGCCTTGTGTTCGTCAGGAAGCC
	T-LF	TGGGGTTGTCTTGCCAAGT
	T-LB	CACCCCGACGAACTCAGT

EBOV: Zaire Ebola virus; SUDV: Sudan Ebola virus; BDBV: Bundibugyo Ebola virus; TAFV: Tai Forest Ebola virus.

**Table 2:** Comparison of the sensitivities in the tube and on the chip.

Sensitivity (Copy Number per Reaction)	EBOV	SUDV	BDBV	TAFV
Tube	100	10	1000	10
Chip	100	1	1000	10

**Table 3:** Test of the specificities of the microfluidic chip to the Ebola virus species.

Numbers of Bio-Reactor Cells	EBOV 6,10,14	SUDV 7,11,15	BDBV 8,12,16	TAFV 9,13,17	Positive Control 23	Negative Controls Others
EBOV	+	−	−	−	+	−
SUDV	−	+	−	−	+	−
BDBV	−	−	+	−	+	−
TAFV	−	−	−	+	+	−
Negative	−	−	−	−	+	−
